# West Nile Virus Subgenomic RNAs Modulate Gene Expression in a Neuronal Cell Line

**DOI:** 10.3390/v16050812

**Published:** 2024-05-20

**Authors:** Maria Bampali, Adamantia Kouvela, Nikolaos Kesesidis, Katerina Kassela, Nikolas Dovrolis, Ioannis Karakasiliotis

**Affiliations:** Laboratory of Biology, Department of Medicine, Democritus University of Thrace, 68100 Alexandroupolis, Greece; mbampali@med.duth.gr (M.B.); kouvela.a@upatras.gr (A.K.); nkesesidis@gmail.com (N.K.); katkassela@gmail.com (K.K.); ndovroli@med.duth.gr (N.D.)

**Keywords:** West Nile virus, subgenomic flaviviral RNA, next-generation sequencing, differential expression analysis, pathway enrichment analysis

## Abstract

Subgenomic flaviviral RNAs (sfRNAs) are small non-coding products of the incomplete degradation of viral genomic RNA. They accumulate during flaviviral infection and have been associated with many functional roles inside the host cell. Studies so far have demonstrated that sfRNA plays a crucial role in determining West Nile virus (WNV) pathogenicity. However, its modulatory role on neuronal homeostasis has not been studied in depth. In this study, we investigated the mechanism of sfRNA biosynthesis and its importance for WNV replication in neuronal cells. We found that sfRNA1 is functionally redundant for both replication and translation of WNV. However, the concurrent absence of sfRNA1 and sfRNA2 species is detrimental for the survival of the virus. Differential expression analysis on RNA-seq data from WT and ΔsfRNA replicon cell lines revealed transcriptional changes induced by sfRNA and identified a number of putative targets. Overall, it was shown that sfRNA contributes to the viral evasion by suppressing the interferon-mediated antiviral response. An additional differential expression analysis among replicon and control Neuro2A cells also clarified the transcriptional changes that support WNV replication in neuronal cells. Increased levels of translation and oxidative phosphorylation, post-translational modification processes, and activated DNA repair pathways were observed in replicon cell lines, while developmental processes such as axonal growth were deficient.

## 1. Introduction

West Nile virus (WNV) is a widely spread neurotropic flavivirus that has emerged as one of the most common causes of encephalitis in the world. It belongs to the *Flavivirus* genus (*Flaviviridae* family) along with other pathogens such as dengue virus (DENV), Zika virus (ZIKV), yellow fever virus (YFV), Japanese encephalitis virus (JEV), and tickborne encephalitis virus (TBEV) [1]. Most of the people infected with the virus remain asymptomatic (80%) or develop mild flu-like symptoms such as fever, headache, myalgia, and fatigue (20%) [2,3]. However, in fewer than 1% of the cases, the virus will cause severe neurological disease, which manifests as encephalitis, meningitis, or poliomyelitis-like acute flaccid paralysis [2,4]. Unfortunately, there are no antiviral drugs available to treat flavivirus infections, and no candidate vaccine for humans has yet proceeded past phase II in clinical trials [5,6].

WNV is a positive-sense single-stranded RNA (+ssRNA) virus. Its genome is about 11 kb in length. It consists of an open reading frame (ORF), which encodes a single polyprotein that is further processed, co- and post-translationally, into three structural (C, prM/M, E) and seven non-structural proteins (NS1, NS2A, NS2B, NS3, NS4A, NS4B, and NS5) [1]. This ORF is flanked by the 5′ and 3′ untranslated regions (UTRs) that form highly structured domains with regulatory roles [7]. The 5′ UTR is about 100 nucleotides long and contains elements with significant roles in replication and translation [8,9]. The 3′ UTR is relatively larger, reaching approximately 630 nucleotides [10]. It is divided into three highly structured domains. The most conserved of them (Domain III) is located at the very end of the 3′ UTR and consists of two adjacent stem-loops, namely the small hairpin (sHP) and the larger 3′ stem-loop (3′ SL) [11]. Upstream of this highly conserved domain are two regions with more variability among flaviviruses, Domain I (stem-loop domain) and Domain II (dumbbell domain) [8,9]. A common characteristic of these regions is the duplication of specific elements, a pattern that seems to be associated with the process of viral adaptation during the host switch, especially for viruses that infect both mammalian and insect cells [11,12]. Domain I is located just after the translation stop codon and contains an AU-rich stem-loop structure (SL-I) followed by a second one (SL-II) that folds into a pseudoknot (PK1) via its interaction with a downstream complementary sequence. The motif is completed with the presence of a short, conserved hairpin (RCS3). This structural unit is then repeated with SL-III, SL-IV, and CS3 elements. The nucleotides of the apical loop of SL-IV also form a pseudoknot with a complementary sequence immediately downstream (PK2). Domain II contains two copies of a dumbbell structure. A third pseudoknot (PK3) is formed via the interaction of the first dumbbell’s loop (TL1) with a complementary sequence at the end of Domain II, and a fourth pseudoknot (PK4) is formed via the interaction of the second dumbbell’s loop (TL2) with a complementary sequence at the beginning of Domain III [8,13]. The structural elements and the pseudoknot interactions within WNV 3’ UTR are shown in Supplementary Figure S1.

XRN-1 is an enzyme with a key role in the mRNA decay pathway. It recognizes decapped RNAs and starts removing nucleotides in a 5′-to-3′ direction, a process that is usually carried out in the P-bodies or stress granules of the cell. During WNV infection, XRN-1 starts digesting the decapped viral genome. However, the presence of highly ordered structures in the 3′ UTR stalls XRN-1 and inhibits further degradation of the viral genome [14]. This results in the accumulation of small RNAs called subgenomic flaviviral RNAs (sfRNAs) that are associated with many functional roles. Stalling of XRN1 in the SL-II/PK1 structure leads to the accumulation of sfRNA1. In some cases, XRN-1 can slip through the first halt site and stop at the next structure, SL-IV/PK2, leading to the formation of a shorter molecule, namely sfRNA2. Further progress of the exoribonuclease through this block can also happen. This time, an even shorter sfRNA is produced (sfRNA3) due to the halting of the enzyme to the downstream DB1/PK3 structure. It is also speculated that DB2/PK4 can function as a mechanical block for XRN-1 and lead to the formation of sfRNA4 [15].

Studies so far have demonstrated that sfRNA has a crucial role in determining viral pathogenicity [16,17]. Along with other viral and/or cellular factors, sfRNA seems to promote a virus-induced cytopathic effect, both in mammalian and mosquito cells. sfRNA suppresses type I IFN and Toll pathway in vertebrates and mosquitoes, respectively [15]. One possible explanation could be that it blocks the cellular RNA sensors that would normally activate the IFN responses [18]. However, this theory has not been proven yet. It could also act as decoy for some interferon-stimulated genes that would normally target the viral RNA. The mechanism of inhibition could be more general and include other factors such as the ability of sfRNA to serve as a sink for cellular RNA-binding proteins (RBPs) [19,20]. sfRNA interferes also with the RNAi mechanism, the most prominent defense mechanism of invertebrates and plants against viruses [21,22]. sfRNA contains highly ordered structures that resemble the secondary structures of the precursors of the Dicer enzyme substrates. Therefore, it is likely that these molecules act as decoys and sequester Dicer, leading to a downregulation in the natural silencing processes [22]. Another important aspect is that sfRNA reduces the availability of XRN-1, leading to an increase in the stability of many short-lived mRNAs, some of which may be negative regulators of the IFN and RNAi pathways [15].

In this study, we investigated the mechanism of sfRNA biosynthesis and its importance for WNV replication into neuronal cells. Using RNA sequencing, we were able to elucidate the impact of sfRNA on the transcriptional regulation of the cells. Neuro2A cells were transfected with the wild-type (WT) and sfRNA-deficient WNV replicons (ΔsfRNA) in order to assess genes that are targeted by sfRNA during WNV replication and characterize pathways that are modulated by its presence. Moreover, an additional differential expression analysis was also conducted among replicon-carrying Neuro2A cells and control cells in order to elucidate the transcriptome changes that support the efficient replication of WNV.

## 2. Materials and Methods

### 2.1. Construction of ΔsfRNA Replicon Plasmids

The plasmid carrying the wild-type (WT) lineage II WNV replicon (pCMVWRep2aH-REN) was kindly provided by Pierson et al. [23]. Briefly, it is a bicistronic construct encoding the full length of the WNV non-structural proteins, followed by the blasticidin resistance gene which is under the translational control of an IRES. Most of the sequence of its structural genes has been removed and replaced with the 2A autoprotease of foot and mouth disease virus (FMDV) and the reporter gene of Renilla luciferase. All these sequences are engineered under the transcriptional control of the CMV promoter.

**Figure 1:**
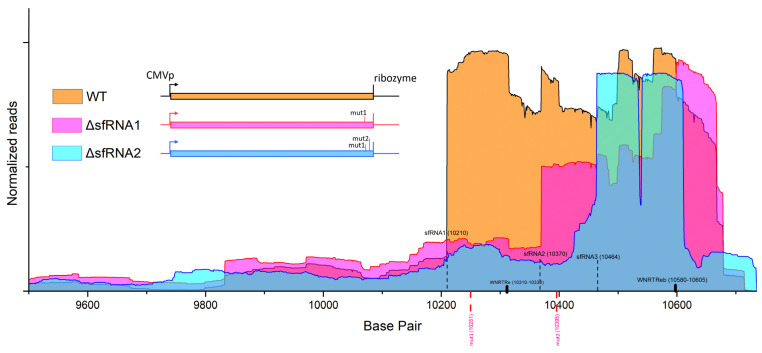
Coverage map of West Nile virus (WNV) replicon 3′ UTR showing the starting points of subgenomic flaviviral RNA (sfRNA) species. Normalized reads per nucleotide position are shown for the 3′ UTR of WT (orange), ΔsfRNA1 (magenta), and ΔsfRNA2 (cyan) replicons. The position of inserted mutations (mut1, mut2) is shown with pink dashed lines. Sharp fluctuation of counts reflects sfRNA start. Black dashed lines indicate the starting points of sfRNAs. The structure of the replicon constructs are also presented as an inset in the figure (WT, ΔsfRNA1, ΔsfRNA2).

The ΔsfRNA constructs were generated by PCR-mediated site-directed mutagenesis with the primers listed in Table S1 (red letters indicate the mutated bases). For the generation of ΔsfRNA1 plasmid, a pair of primers originally designed by Pijlman et al. [16] were used (WNVIRAmutF, WNVIRAmutR) and were slightly modified in order to match our template sequence. These primers incorporated a 3-nucleotide substitution in an IRA (inverted repeats) sequence of SL-II (represented as “mut1” in Figure 1, Supplementary Figure S1). The mutation has been shown to disrupt the SL-II structure and abolish the production of sfRNA1. Two external primers were also designed that enclosed the mutated area into two BstEII restriction sites (WNVIRAextF, WNVIRAextR). At first, two PCR reactions were performed using WNVIRAextF-WNVIRAmutR and WNVIRAmutF-WNVIRAextR primer pairs (20 pmol each) along with 0.5 μg of pCMVWRep2aH-REN plasmid as the DNA template, 1 μL of dNTPs (10 mM), and 0.3 μL of Taq DNA Polymerase (5 U/μL) in a 50 μL PCR reaction mixture, containing 5 μL of Taq Buffer A (10×) from the KAPA Taq PCR Kit (Kapa Biosystems, Cape Town, South Africa). The amplification protocol was as follows: an initial step at 95 °C for 2 min and then at 95 °C for 30 s for template denaturation, at 58 °C for 30 s for primer annealing, and then at 72 °C for 45 s for extension (35 cycles). A final extension step at 72 °C for 5 min was also performed. The PCR products (0.5 and 1 μg, respectively) were then used together as templates and primers for each other in a third PCR reaction, which was performed following the same protocol that is described above. After 5 reaction cycles, the external primers WNVIRAextF and WNVIRAextR (20 pmol each) were also added. The mutated fragment was then digested with BstEII restriction enzyme and was reinserted into the pCMVWRep2aH-REN plasmid that had been cut open with the same enzyme. The presence of mutations was confirmed by Sanger sequencing.

A similar procedure was also followed for the generation of the ΔsfRNA2 construct. A pair of primers were designed (WNVmut10395F, WNVmut10395R) based on the observations of Chapman et al. [24], who showed that a C→G point mutation in one of the unpaired nucleotides within the SL-IV junction in Kunjin genome abolishes the production of sfRNA2 (represented as “mut2” in Figure 1, Supplementary Figure S1). Alignment of Kunjin virus infectious clone (described in [24]) with the replicon sequence revealed its analogous position in the WNV replicon (C10680→G in WNVKUNxrRNA2 and C10395→G in the WNV replicon SL-IV structure). Two external primers were also designed that enclosed the mutated area into two XbaI restriction sites (WNVext10395F, WNVext10395R). At first, two PCR reactions were performed using WNVext10395F-WNVmut10395R and WNVmut10395F-WNVext10395R primer pairs (20 pmol each) along with 0.5 μg of ΔsfRNA1 plasmid as the DNA template, 1 μL of dNTPs (10 mM), and 0.3 μL of Taq DNA Polymerase (5 U/μL) in a 50 μL PCR reaction mixture, containing 5 μL of Taq Buffer A (10×) from KAPA Taq PCR Kit (Kapa Biosystems, Cape Town, South Africa). The amplification protocol was as follows: an initial step at 95 °C for 1 min and then at 95 °C for 30 s for template denaturation, at 55 °C for 45 s for primer annealing, and then at 72 °C for 1 min for extension (40 cycles). A final extension step at 72 °C for 7 min was also performed. The PCR products (0.5 μg) were then used together as templates and primers for each other in a third PCR reaction, which was performed following the same protocol that is described above. After 5 reaction cycles, the external primers WNVext10395F and WNVext10395R (20 pmol each) were also added. The mutated fragment was then digested with XbaI restriction enzyme and was reinserted into the ΔsfRNA1 pCMVWRep2aH-REN plasmid that had been cut open with the same enzyme. The presence of mutations was confirmed by Sanger sequencing.

### 2.2. Cell Cultures

Neuro2A cells were kindly provided by Dr. Rebecca Matsas (Hellenic Pasteur Institute, Athens, Greece). They were cultured in Dulbecco’s modified Eagle medium (DMEM) supplemented with 10% fetal bovine serum (FBS) and 1% PenStrep (100 U/mL Penicilium and 100 μg/mL Streptomycin). The cells were maintained in a humidified incubator at 37 °C under 5% CO_2_.

### 2.3. Translation Efficiency of WT and ΔsfRNA Replicons

Neuro2A cells were transfected with the plasmids carrying the WT and ΔsfRNA replicons using jetPRIME^®^ transfection reagent (Polyplus Transfection, Illkirsch, France). Then, luciferase activity measurements were performed in order to evaluate the translation efficiency of replicons over the course of four days. In brief, cells were harvested at 6, 24, 36, 48, 72, and 96 h post-transfection and lysed in freshly prepared Passive Lysis Buffer (Promega Corp., Madison, WI, USA). Coelenterazine substrate (Promega Corp.) was prepared as a 1 mg/mL stock solution in acidified methanol and then diluted to 1:500 in Renilla buffer (50 mM K_3_PO_4_ pH:7.5, 500 mM NaCl, 1 mM EDTA). At the time of the measurement, cell lysates were mixed with coelenterazine. Luminescence from Renilla luciferase activity was measured for 10 s in a Varioskan™ LUX multimode microplate reader (Thermo Fisher Scientific, Waltham, MA, USA). Cells were also co-transfected with a plasmid encoding Firefly luciferase, whose expression was used as a normalizer. In this case, cell lysates were mixed with Luciferase Assay Substrate (Promega Corp.). The background signal was assessed by measuring luciferase activity from mock cells and subtracted from the samples’ readings. All transfections and luciferase measurements were performed in triplicate.

### 2.4. RNA Extraction and qPCR for the Estimation of Replication Efficiency

Neuro2A cells were transfected with the plasmids carrying the WT and ΔsfRNA replicons using jetPRIME^®^ transfection reagent (Polyplus Transfection). At 48 h post-transfection, cells were harvested, and total RNA was isolated using NucleoZOL (Macherey-Nagel, Düren, Germany) according to the manufacturer’s instructions. RNA was first treated with DNase (Promega Corp.) for 30 min in order to remove any contaminating DNA and then was converted to cDNA with MMLV reverse transcriptase (Promega Corp.) and primers specific for the viral NS1 gene (Table S1). The resulting cDNA was then used as a template in a real-time PCR analysis in order to assess the abundance of replicon RNA. qPCR analysis was performed using with KAPA SYBR^®^ FAST Universal (Kapa Biosystems, Cape Town, South Africa). The amplification protocol was as follows: an initial step at 95 °C for 10 min and then at 95 °C for 20 s for template denaturation, at 60 °C for 20 s for primer annealing, and at 72 °C for 30 s for extension (40 cycles). Real-time PCR reactions were carried out in a Stratagene Mx3000P Real-time PCR system, set to record fluorescence for SYBR Green I (520 nm). Each sample was run in triplicate, and Ct values were collected and used for the subsequent analyses. In order to normalize the viral expression levels, the *ΥWHAΖ* housekeeping gene was also tested. Primers for *YWHAZ* were obtained from Primer Bank (PrimerBank ID: 6756041a1) [25].

### 2.5. Generation of the WT and ΔsfRNA Stable Cell Lines

Neuro2A cells were transfected with the plasmids carrying the WT and ΔsfRNA replicons using jetPRIME^®^ transfection reagent (Polyplus Transfection). At 48 h post-transfection, blasticidin S antibiotic (PanReac AppliChem, Darmstadt, Germany) was added to the culture medium at a concentration of 10 μg/mL. The treatment was continued for a month. Renilla luciferase activity was finally measured in order to check for the expression of replicons in the surviving cells.

### 2.6. Affinity Enrichment Protocol

Total RNA was extracted from WT and ΔsfRNA stable cell lines with Trizol (Invitrogen Life Technologies, Thermo Fisher Scientific, Waltham, MA, USA). The isolated RNA was then mixed with biotin-modified probes specific for WNV 3′ UTR (BIO-WNRTRe, BIO-WNRTReb) in order to capture the viral sfRNA species. The probes are listed on Table S1. After that, the sfRNA–probe complexes were immobilized on streptavidin beads (New England Biolabs, Ipswich, MA, USA), and the unwanted cellular RNA was removed. These sfRNA-enriched samples were then sequenced.

### 2.7. RNA Sequencing of sfRNA

Barcoded libraries were prepared for the isolated sfRNAs using the Ion Total RNA—Seq v2 Core Kit (Thermo Fisher Scientific) according to the manufacturer’s instructions. In brief, RNA was reverse transcribed into a library of cDNA molecules that were later amplified through a PCR reaction with the simultaneous attachment of sample-specific barcodes from the Ion Xpress^TM^ RNA-Seq Barcode 1-16 Kit (Thermo Fisher Scientific). The median product size of these libraries was evaluated in a LabChip^®^ GX Touch^TM^ Nucleic Acid Analyzer (PerkinElmer, Waltham, MA, USA), and their concentrations were measured via Qubit^TM^ 4 Fluorometer (Thermo Fisher Scientific). The samples were loaded on Ion 540^TM^ chip using the Ion Chef^TM^ instrument, and the sequencing was performed on an Ion GeneStudio S5 System (Thermo Fisher Scientific). The output averaged ~10 million quality-controlled reads per sample with a median read length of 131 bp. The resulting reads were aligned to the WNV replicon reference genome (pCMVWRep2aH-REN) with the Burrows–Wheeler Aligner (BWA) v. 0.7.17-r1188 [26]. Aligned read files were indexed, and the depth per base pair was calculated using SAMtools v.1.11 [27]. Each sample’s read count was normalized to the total number of reads. Then, using Integrative Genomics Viewer (IGV) v.2.5.3 [28], we counted and visualized all reads corresponding to the same nucleotide position. The location of the inserted mutations was also validated with IGV.

### 2.8. Estimation of the Replicon and sfRNA Abundance in Stable Cell Lines

For the determination of the replicon RNA levels in each stable cell line, a real-time PCR analysis was performed. Briefly, total RNA was isolated using NucleoZOL (Macherey-Nagel, Düren, Germany) and treated with DNase (Promega Corp.) for 30 min in order to remove any contaminating genomic DNA. Then, RNA was converted to cDNA with MMLV reverse transcriptase (Promega Corp.) and primers specific for the viral NS1 gene (NS1RT4), targeting both the positive and the negative strand (Table S1). qPCR analysis was subsequently performed with KAPA SYBR^®^ FAST Universal (Kapa Biosystems, Cape Town, South Africa). The amplification protocol and the subsequent procedure for the estimation of the relative abundance were the same as those described at Section 2.4. Another real-time PCR reaction was also conducted to quantify the levels of sfRNA in each stable line. In this case, RNA from each replicon cell line was treated with DNase (Promega Corp.) and then converted to cDNA using a reverse primer (WNVsfR) that hybridizes in a region that is common to all sfRNA species (Supplementary Figure S2). The resulting cDNA was then used as a template in an RT-qPCR analysis, which was performed using primers targeting the aforementioned region (WNVsfF, WNVsfR). The sfRNA-specific primers (WNVsfF, WNVsfR) are listed in Table S1. The amplification protocol and the subsequent procedure for the estimation of the relative abundance were the same as those described in Section 2.4. Viral NS1 gene expression was used for the normalization of the results.

### 2.9. RNA-Seq and Differential Expression Analysis

Total RNA was isolated from WT, ΔsfRNA1, and ΔsfRNA2 stable lines using NucleoZOL (Macherey-Nagel, Düren, Germany). Then, 3′ end libraries were generated with QuantSeq 3′ mRNA-Seq Library Prep Kit FWD (Lexogen, Vienna, Austria). Briefly, RNA was reverse transcribed into cDNA through oligo-dT priming that was specifically designed to select only poly-A RNAs. The RNA strand was then removed, and a second DNA strand was synthesized. The resulting library was amplified with PCR and quantified via Qubit^TM^ 4 Fluorometer (Thermo Fisher Scientific). The median product size of these libraries was also evaluated in a LabChip^®^ GX Touch^TM^ Nucleic Acid Analyzer (PerkinElmer). The libraries were loaded on an Ion 540^TM^ chip using the Ion Chef^TM^ instrument. Sequencing was carried out on Ion GeneStudio S5 System (Thermo Fisher Scientific). The output averaged ~10 million quality-controlled reads per sample with a median read length of 250 bp. Reads were aligned on the mouse transcriptome reference using the Burrows–Wheeler aligner (BWA) v. 0.7.17-r1188 [26]. Aligned read files were indexed, and the depth per base pair was calculated using SAMtools v.1.11 [27]. Reads were quantified using Salmon v.1.4.0 [29] and imported into R v.4.1.0 [30] with the package tximport v.1.28.0 [31]. All differential expression comparisons were conducted using the package DESeq2 v.1.40.2 [32] with the default parameters. The same procedure was also followed for control Neuro2A cells whose transcriptional profile was also compared to that of the WT and ΔsfRNA replicon cell lines.

### 2.10. Pathway Enrichment Analysis of the Differentially Expressed Genes

Differentially expressed genes between the WT and ΔsfRNA cell lines with an adjusted *p*-value < 0.05 and fold change more than +/−2 underwent enrichment analysis via the use of Reactome database [33], with the clusterProfiler R package v.4.8.3 [34] being used to investigate their involvement in biological pathways. Pathway enrichment results were also obtained for the differentially expressed genes among replicon cell lines and control Neuro2A cells using the same methodology.

### 2.11. Validation of the RNA-Seq Results

Validation of the differentially expressed genes was completed with qPCR. Total RNA isolated from WT, ΔsfRNA1, and ΔsfRNA2 stable lines was converted to cDNA using MMLV reverse transcriptase (Promega Corp.) and oligo(dT) 18-mer primer (New England Biolabs, Ipswich, MA, USA). qPCR analysis was performed with KAPA SYBR^®^ FAST Universal (Kapa Biosystems). In total, 25 genes were selected for qPCR based on their involvement in significant biological processes that resulted from the enrichment analysis. The primers are listed in Table S2. All primers were provided by PrimerBank [25], except for the ND1, ND2, and ND4 primers, which were designed by El-Merhie et al. [35]. The amplification protocol was as follows: an initial step at 95 °C for 3 min and then at 95 °C for 20 s for template denaturation, at 60 °C for 20 s for primer annealing, and at 72 °C for 30 s for extension (40 cycles). Real-time PCR reactions were carried out in a Stratagene Mx3000P Real-time PCR system, set to record fluorescence for SYBR Green I (520 nm). All reactions were run in triplicate. Calculation of the relative gene expression was conducted via the 2^−ΔΔCT^ method, with *YWHAZ* being used as the endogenous control.

### 2.12. Measurement of the Interferon Signaling Induction

pISRE-luc plasmid (Stratagene, La Jolla, CA, USA) was transfected into Neuro2A replicon cell lines in order to assess their levels of IFN signaling. The plasmid carries the Firefly luciferase reporter gene under the transcriptional control of an interferon-stimulated response element (ISRE) promoter. Cells were harvested and lysed at 48 h post-transfection. Then, luminescence was measured using Luciferase Assay System (Promega Corp.). The background signal was subtracted, and normalization was performed using Bradford Protein assay (BioRad, Hercules, CA, USA). All experiments were performed independently three times. Neuro2A cells were also treated with conditioned medium containing IFN that was secreted from poly:IC-transfected cells 2 h after pISRE-luc plasmid transfection to test their ability to correspond to it. After 48 h, cells were harvested and lysed, and luminescence was measured.

### 2.13. Calculation of Viral Titer Produced in the WT and ΔsfRNA Replicon Cell Lines

WT, ΔsfRNA1, and ΔsfRNA2 replicon cells, along with plain Neuro2A, were infected with a WNV lineage II strain at a multiplicity of infection (MOI) of 1 in 2% FBS complete DMEM. After 4 h, the medium was replaced with fresh complete DMEM in order to remove the remaining viral particles. Cells were maintained at 37 °C (5% CO_2_) in a humidified environment for two days. At 48 h post-infection, culture media with the newly formed viral particles were harvested. Then, 10-fold serial dilutions were performed into Vero cells and were inoculated for 72 h until viral infection could be determined by the observation of an induced cytopathic effect. Taking into account the lowest dilutions with noticeable signs of viral infection, we calculated the titer of virus (TCID50/mL) produced from each cell line using the Spearman and Kärber algorithm as described by Hierholzer and Killington [36].

## 3. Results

### 3.1. Locating sfRNA Starting Points on WNV Replicon Genome

**Figure 2:**
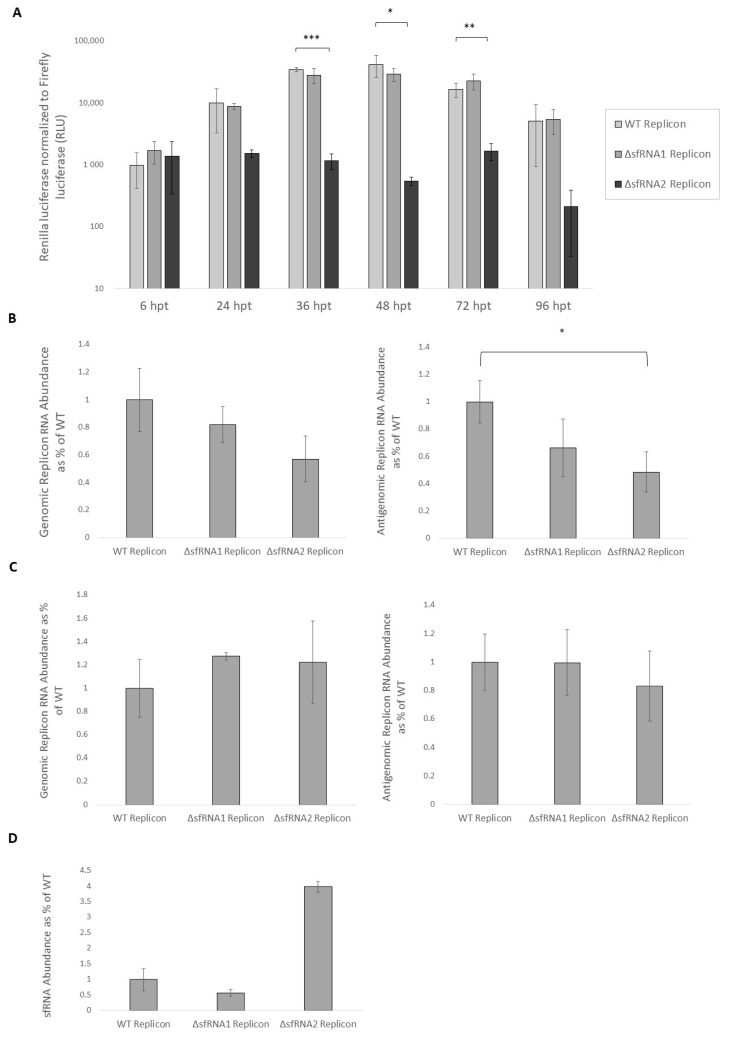
Translation and replication efficiency of WT and ΔsfRNA replicons in Neuro2A cells. (**A**) Comparison of the translation efficiency for WT and ΔsfRNA replicons. Renilla luciferase measurements are indicative of their translation capability. Luciferase was measured over the span of four days at indicated time points. The expression was normalized to that of a co-transfected Firefly luciferase plasmid. (**B**) Relative abundance of the genomic and antigenomic replicon RNA of WT and ΔsfRNA replicons at 48 h post-transfection. *YWHAZ* expression was used for normalization. (**C**) Relative abundance of the genomic and antigenomic replicon RNA in the WT and ΔsfRNA stable replicon cell lines. (**D**) Relative abundance of sfRNA in the WT and ΔsfRNA stable replicon cell lines. Viral NS1 gene expression was used for normalization. Error bars correspond to the standard deviation from 3 technical replicates. * *p* < 0.05, ** *p* < 0.01, *** *p* < 0.001 (Student’s *t* test).

sfRNA is the product of XRN-1 stalling on secondary structures in the flaviviral genome. In our study, we inserted mutations in specific sites that have been demonstrated to disrupt the formation of these XRN-1 inhibitory structures in order to select the smaller sfRNA species and identify, via next-generation sequencing, their 5′ ends on the viral genome (ΔsfRNA replicons). These plasmids were transfected into Neuro2A cells. WT replicon construct predominantly produced sfRNA1, ΔsfRNA1 produced sfRNA2, and ΔsfRNA2 produced sfRNA3. Through an affinity enrichment protocol, these sfRNA species were then selected and sequenced in order to localize their precise positions on the viral 3′ UTR. The reads of each nucleotide position were counted. The results are depicted in Figure 1. Sharp fluctuation of counts reflects the sfRNA start. In this way, we accurately determined the exact starting points of WNV sfRNAs on the viral genome and also estimated their sizes to be 512 bp (sfRNA1), 365 bp (sfRNA2) and 271 bp (sfRNA3). The sudden decrease in the base count of sfRNA3 3′ end was probably an artifact. A qPCR analysis showed that the corresponding region was, in fact, present in all sfRNA species (described in Section 2.8; the results are shown in Figure 2D).

### 3.2. Translation and Replication Efficiency of the WT and ΔsfRNA Replicons

In order to assess the importance of sfRNA for viral translation, we measured the activity of Renilla luciferase, the reporter gene of the WT and ΔsfRNA replicons, when transfected into Neuro2A cells. At 6, 24, 36, 48, 72, and 96 h post transfection, cells were harvested and lysed. As shown on Figure 2A, the absence of sfRNA1 did not have an important reduction in the translation efficiency of ΔsfRNA1 replicon, while the absence of both sfRNAs significantly depleted the process of viral protein synthesis. At the time when replicons presented their highest expression levels (48 h post-transfection), RNA was also extracted from the WT and ΔsfRNA cell lines in order to assess the abundance of replicon RNA via an RT-qPCR analysis (Figure 2B). A significant reduction in the synthesis of the negative strand was observed when both sfRNAs were absent. It must be noted that the inserted mutations did not inherently attenuate the processes of translation or replication since stable lines with high expression levels of their replicons were also developed. These results indicate that the absence of sfRNA1 is well tolerated by WNV and compensated for by sfRNA2, but when both sfRNA1 and sfRNA2 are missing, the virus presents attenuated phenotype regarding its replication and translation levels.

### 3.3. Differential Expression Analysis among the WT and ΔsfRNA Replicon Cell Lines

**Figure 3:**
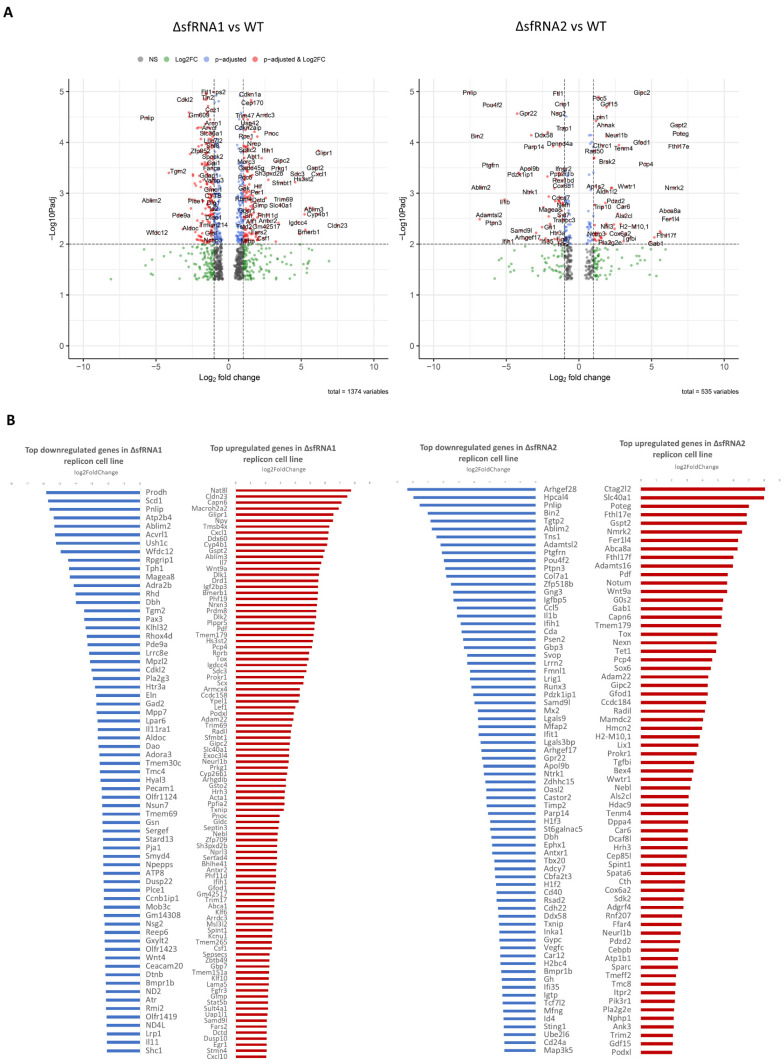

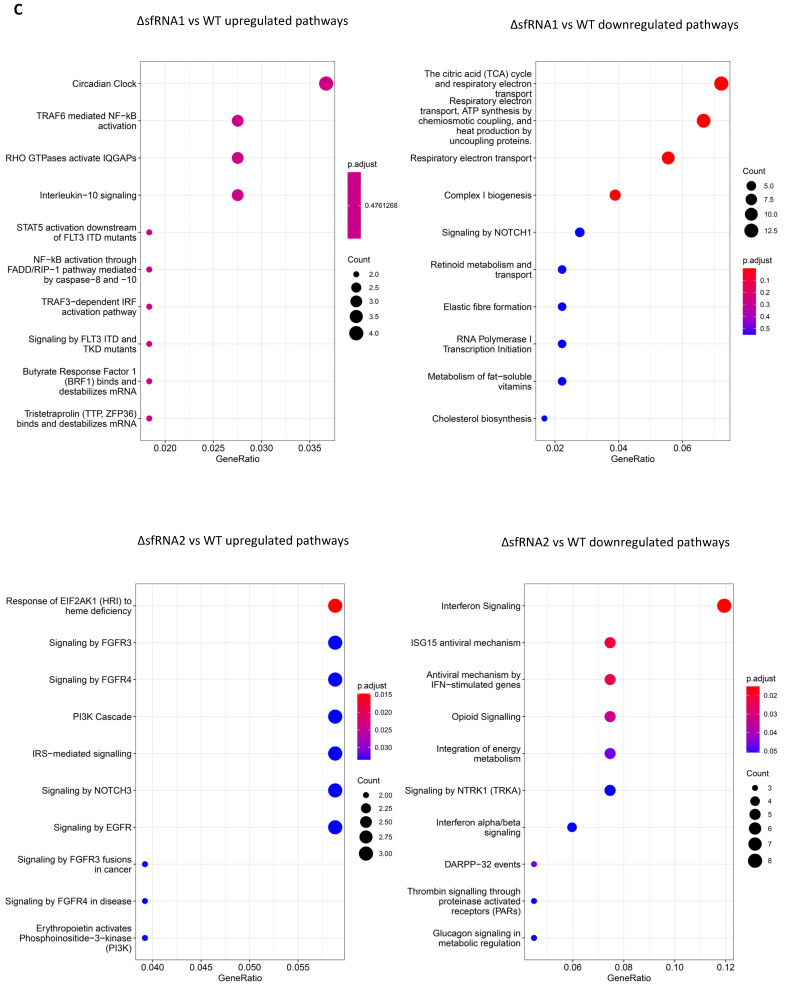

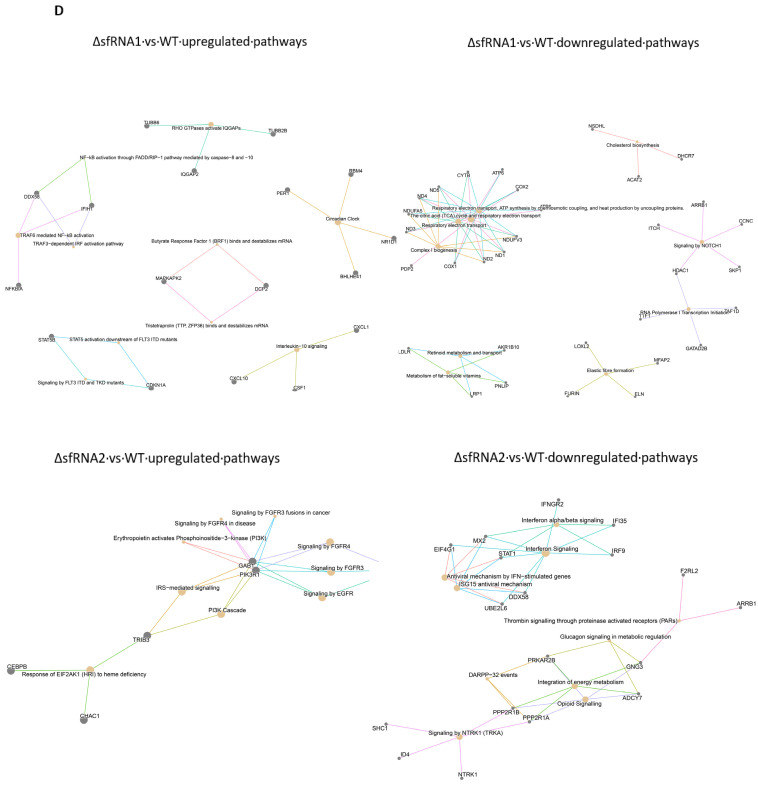

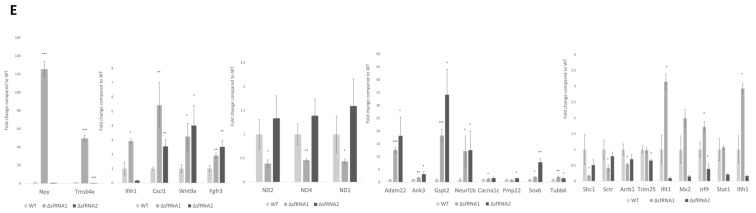
Differentially regulated genes and altered pathways among the WT and ΔsfRNA replicon cell lines. (**A**) Differentially expressed genes in the ΔsfRNA1 (**left** image) and ΔsfRNA2 (**right** image) replicon cell lines plotted as volcano plots. (**B**) Top downregulated and upregulated genes of the ΔsfRNA1 and ΔsfRNA2 replicon cell lines when compared against the WT replicon cell line. (**C**) Pathway enrichment analysis results for upregulated and downregulated genes in the ΔsfRNA1 and ΔsfRNA2 replicon cell lines. (**D**) Cnetplot depicting the top 10 upregulated and downregulated Reactome pathways in ΔsfRNA1 and ΔsfRNA2 cells as networks that connect the genes with the respective pathways. (**E**) qPCR validation results for a selected set of genes. Error bars correspond to the standard deviation from 3 technical replicates. * *p* < 0.05, ** *p* < 0.01, *** *p* < 0.001 (Student’s *t* test).

By applying continuous selective pressure via the administration of antibiotic, we created stably expressing WT and ΔsfRNA replicon cell lines, allowing for the replication of the mutated replicons. As shown on Figure 2C, comparable levels of replicon RNA expression were observed for the WT and ΔsfRNA cell lines. sfRNA abundance was also estimated with a primer pair that binds to a region in the 3′ end that is common to all sfRNA species (Figure 2D, Supplementary Figure S2). NGS of the replicons that were isolated from these stable lines revealed no adaptive mutations in their sequence that would explain the elevated levels of replication, especially for ΔsfRNA2 replicon. Thus, we hypothesized that the selected cells provided a more favorable environment instead. In order to identify the differentially regulated pathways that created this environment, an RNA sequencing analysis was conducted. By comparing the WT and ΔsfRNA cell lines, a list of differentially expressed genes was generated (Figure 3A,B). The complete list is provided in Supplementary Tables S3–S6. A total of 331 genes were significantly upregulated in ΔsfRNA1 replicon cells compared to WT replicon cells by 2 folds or more (adjusted *p*-value < 0.05), while 167 genes were significantly upregulated in ΔsfRNA2 replicon cells. On the other hand, 398 genes were significantly downregulated in ΔsfRNA1 replicon cells by 2 folds or more (adjusted *p*-value < 0.05), and 147 genes were significantly downregulated in the ΔsfRNA2 replicon cells.

### 3.4. Pathway Enrichment Analysis for Differentially Regulated Genes among the WT and ΔsfRNA Replicon Cell Lines

Differentially expressed genes with more than +/−2-fold change underwent enrichment analysis via Reactome to investigate their involvement in biological pathways. The most significant of them are presented on Figure 3C,D. Extended lists of the top 25 enriched pathways in each category are also provided in Supplementary Tables S7–S11. The expression of 25 genes was also tested with qPCR for the validation of the NGS analysis. Results are presented on Figure 3E (also Supplementary Tables S12 and S13). Generally, qPCR results were in good accordance with RNA sequencing.

Circadian clock pathways and interleukin signaling were the most enriched in genes with upregulated expression in the ΔsfRNA1 replicon cell line (Figure 3C,D). Pro-inflammatory cytokine Il7 and chemokines Cxcl1 and Cxcl10 were positively regulated in the absence of sfRNA1. The Ddx58 (RIG-I) and Ifih1 gene (MDA5) were also upregulated (Figure 3B,E). Other positively regulated genes were Npy, Tmsb4x, Fgfr3, and Wnt9a (Figure 3B,E).

On the other hand, the ΔsfRNA1 replicon cell line had a significantly lower energy metabolism and lipid metabolism (cholesterol biosynthesis, retinoid metabolism, and transport) in comparison with the WT replicon line (Figure 3C,D). Downregulated gene sets were enriched in molecules implicated in TCA cycle and oxidative phosphorylation (Nd1-Nd6, Atp6, Atp8, Ndufv3, Ndufa5, Cox1-Cox3, Cytb) (Figure 3B,E). Notch signaling was also found to be dysregulated. In fact, many negative regulators of Notch signaling were found to be downregulated in the ΔsfRNA1 cell line (Skp1, Itch, Arrb1, Hdac1). All these proteins mediate the ubiquitination and degradation of Notch.

In the ΔsfRNA2 replicon cell line, receptor tyrosine kinase signaling (FGFR/EGFR signaling, PI3K cascade) was among the top enriched pathways in genes with upregulated expression (Gab1, Fgfr3, Pik3r1) (Figure 3B–E). Besides this, more genes related to immune and inflammatory responses were upregulated in the absence of the inhibitory regulation of both sfRNA1 and sfRNA2. Transcription factor Cebpb was upregulated. CEBPB regulates, among others, the expression of genes involved in immune and inflammatory responses such as cytokines IL-6, IL-4, IL-5, and TNF-alpha. Tet1, which regulates the transcription of TNF-alpha and the activation of macrophages, was also upregulated. Genes related to Ca^2+^ signaling (Capn6, Camk4, Pcp4) were also found to be significantly upregulated. Mamdc2 overexpression was also observed. Such an overexpression has been previously shown to enhance the innate antiviral response in mouse brains during neurotropic virus infection [37]. Prokr1 was another gene with positive regulation. Prokr1 is a G-protein-coupled receptor that, along with its ligands, prokineticins, has been associated with inflammation. Mib2 and Neurl1, two E3 ubiquitin protein ligases that are involved in the negative regulation of Notch signaling pathway, were also upregulated. Ank3 and Cacna1c, which have been associated with channelopathies and the development of neuropsychiatric disorders, also presented an upregulation in their expression levels [38,39]. Besides Ank3, another ankyrin with very high expression levels in the ΔsfRNA2 replicon cell line was POTEG. Gspt2 pro-apoptotic factor, Adam metalloproteinases (Adam22, Adamts16), and Slc40a1 ferroportin were also among the most upregulated genes in the ΔsfRNA2 replicon cell line. In fact, these genes presented significantly decreased levels in the WT replicon cell line that recovered in the absence of the inhibitory regulation of sfRNA1 and sfRNA2 (Figure 3B,E).

Nevertheless, ΔsfRNA2 replicon cells generally presented an inherent impairment in their IFN activity that compensated for the absence of the protective role of sfRNAs. A great number of genes implicated in the interferon signaling pathway were significantly downregulated (Figure 3). In fact, the inhibition of the innate immunity response seems to be a prerequisite for the efficient propagation of the double-mutant replicon.

### 3.5. Differential Expression Analysis among Replicon and Control Neuro2A Cell Lines

**Figure 4:**
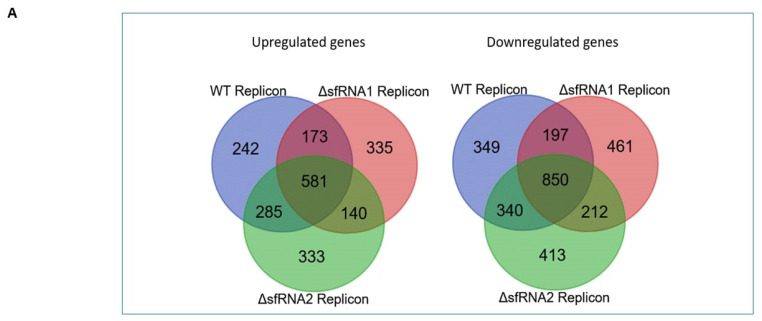

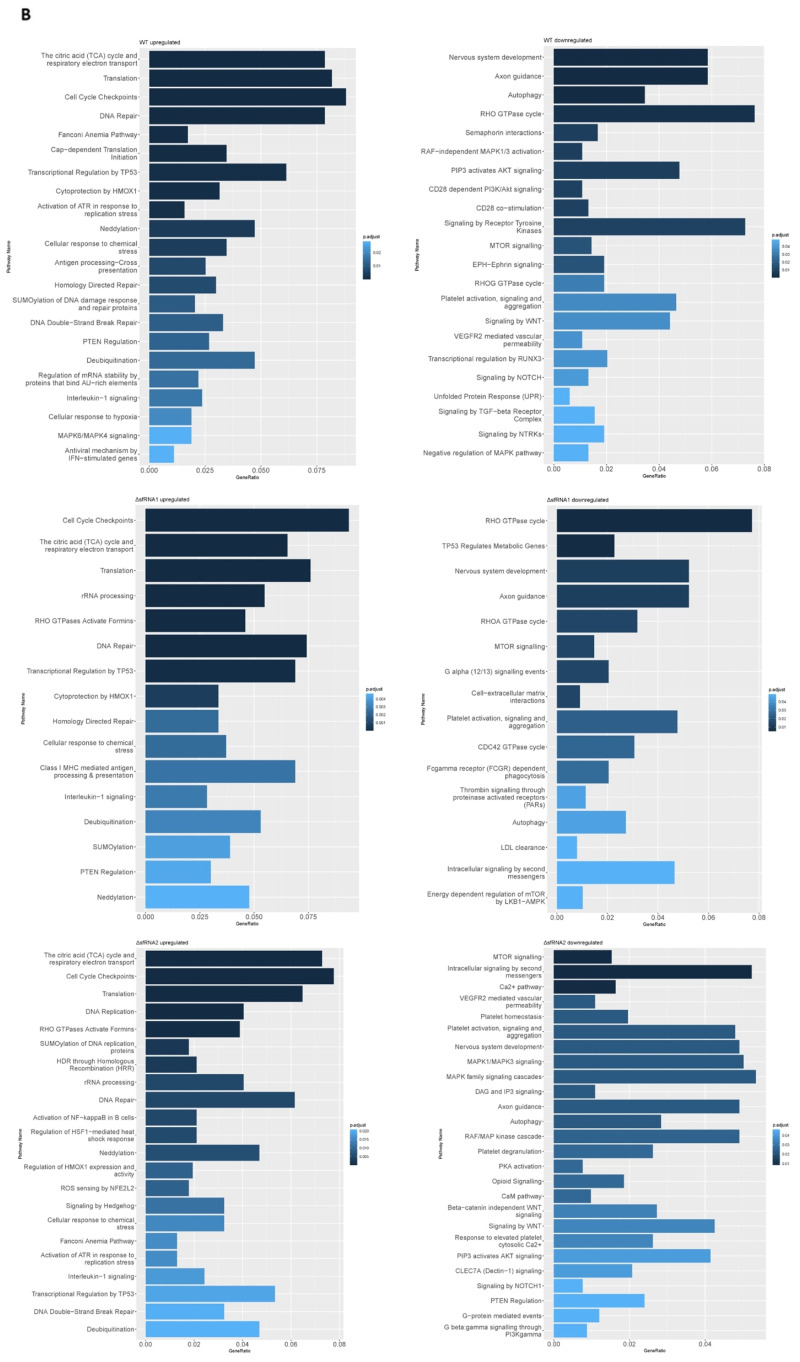
Differentially regulated genes and altered pathways among the replicon and control Neuro2A cell lines. (**A**) Venn diagram showing the number of common upregulated and downregulated genes among the WT, ΔsfRNA1, and ΔsfRNA2 replicon cells when compared with the control Neuro2A cell lines. (**B**) Reactome plots depicting the results from pathway enrichment analysis for the upregulated and downregulated genes in each category.

An additional differential expression analysis was also conducted among replicon cell lines (WT and ΔsfRNA) and control Neuro2A cells in order to identify transcriptional changes in the selected stable lines that support WNV replication. A total of 1280 genes were significantly upregulated in the WT replicon cells compared to control cells by 2 folds or more (adjusted *p*-value < 0.05), 1228 genes in ΔsfRNA1 replicon cells, and 1338 in ΔsfRNA2 cells. On the other hand, 1736 genes were significantly downregulated in WT replicon cells by 2 folds or more (adjusted *p*-value < 0.05), 1719 in ΔsfRNA1 replicon cells, and 1815 in ΔsfRNA2 cells (Supplementary Tables S14–S19). The Venn diagram in Figure 4A shows the number of common upregulated and downregulated genes among the three groups. Genes with more than +/−2-fold change underwent enrichment analysis so that their involvement in biological pathways could be examined.

### 3.6. Pathway Enrichment Analysis for the Differentially Regulated Genes among the Replicon and Control Neuro2A Cell Lines

The most significantly enriched biological pathways are presented on Figure 4B. Genes implicated in ATP synthesis via TCA cycle and oxidative phosphorylation were significantly upregulated in all replicon cell lines. The translation and mitochondrial translation pathways were also upregulated. The ubiquitin/proteasome system was also positively regulated. Many proteasome subunits (Psmd1, Psme1, Psmc2, Psmd5, Psmb6, Psmb8, Psmb10, Psmd12, Psme3) were included in the gene lists, along with ubiquitin (Ubb). A number of genes encoding deubiquitylating enzymes (Uchl3, Bap1, Usp16, Abraxas2, Otud3) were also found to be upregulated in the replicon cell lines. Usp18, especially, was significantly overexpressed in the WT and ΔsfRNA1 replicon cell lines. Similar processes that involve the post-translational conjugation of ubiquitin-like modifiers to target proteins also emerged from the enrichment analysis. Neddylation (Cul1, Cops5, Cops2, Cops7a, Ube2f, Lrr1, Dtl, Spsb4, Asb8, Rnf7, Klhl25) and SUMOylation processes (Trim27, Uba2) were significantly enriched. Cell cycle checkpoints/DNA repair processes were also positively regulated in the replicon cell lines.

On the other hand, nervous system development, axon guidance, and autophagy were significantly downregulated in the replicon cell lines. The mTOR signaling pathway’ s positive regulators (Akt2, Lamtor1, Mlst8, Cab39, Slc38a9, Eif4g1, Eif4b, Rraga) and negative regulators (Stk11/Lkb1, Prkag2, Prkab2, Redd1/Ddit4, Deptor, Sestrin2, Sestrin3) were found among the downregulated gene lists. Replicon cell lines presented also a downregulation in the expression of a number of Rho GTPases (RhoV, RhoQ, RhoC, RhoA, RhoU), other G protein-signal transducers (Gng3, Gng4, Gnb2, Gna12, Gnb3, Gna11, Gnl3l, Gnb4), and related regulatory proteins (Arhgef28, Arhgap27, Arhgap18, Arhgef17, Arhgef25, Arhgef19, Arhgdib, Arhgap31, Arhgef39, Arhgap12, Fgd1, Prex2, Cdc42ep1, Cdc42ep2, Prex1, Gmip, Dock6, Iqgap1, Fgd1, Srgap3). Proteins of this family are responsible for the regulation of the movement of the cell and cytoskeletal dynamics. Other factors that regulate intracellular activity of Rho GTPases such as semaphorins and ephrins were also found to be downregulated (Sema6c, Sema4a, Sema4d, Sema4b, Efna2), as were calmodulins Calm1 and Calm2, which have been shown to regulate GTPase signaling. All pathways from the enrichment analysis are also included in Supplementary Table S20.

### 3.7. The Modulatory Role of sfRNA in the Innate immunity Pathway

**Figure 5:**
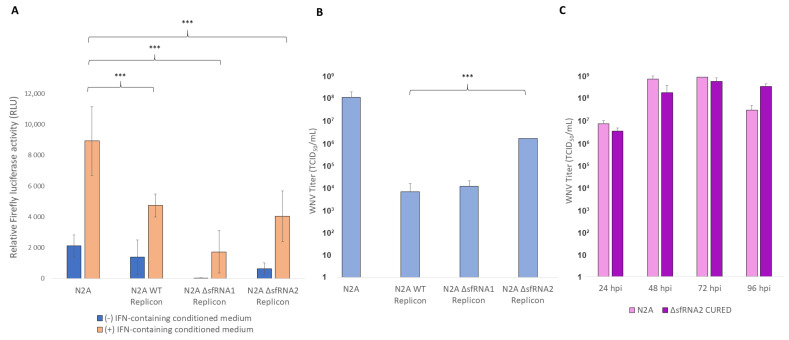
Induction of interferon signaling in the Neuro2A WT and ΔsfRNA replicon cell lines. (**A**) Luciferase measurements from the ISRE plasmid in the Neuro2A WT and ΔsfRNA cell lines. Cells were tested under normal conditions (-IFN-containing conditioned medium) or after their treatment with conditioned medium containing IFN secreted from poly:IC-transfected cells (+IFN-containing conditioned medium). Luciferase activity is indicative of the cell’s IFN competency. Error bars correspond to the standard deviation from 3 biological replicates. *** *p* < 0.001 (Student’s *t* test). (**B**) Comparison of the WNV titer produced from the Neuro2A WT and ΔsfRNA replicon cell lines at 48 hpi. *** *p* < 0.001 (Student’s *t* test). (**C**) WNV titer produced in the Neuro2A ΔsfRNA2 CURED cell line in comparison with that of the plain Neuro2A cells at indicated time points.

One of the most significant observations from the above analyses was that sfRNA conferred protection against the host’s IFN signaling. When sfRNA1 was absent, an upregulation in the expression of specific innate immunity-related genes was observed, and when both sfRNA1 and sfRNA2 were not produced, differential expression analysis showed an upregulation in a broader range of antiviral genes (Figure 3). In fact, the double mutant replicon was only able to survive in inherently IFN-incompetent cells. In order to verify the competence of the cell lines to respond to IFN signaling, a luciferase-reporter gene assay was applied. A plasmid carrying the Firefly luciferase reporter gene under the transcriptional control of an interferon-stimulated response element (ISRE) promoter was used. Normally, type I or III IFNs bind to their cell-surface receptors and activate the JAK/STAT signaling cascade. Activated STATs then form ISGF3 complex together with IRF9 and bind to ISRE-containing promoters that are usually present in IFN-α/β inducible genes. This assay estimates the induction of interferon signaling indirectly by measuring the expression of the Firefly luciferase gene. The plasmid was transfected into Neuro2A WT and ΔsfRNA replicon cell lines, as well as control Νeuro2A cells, and luciferase activity was measured. The same cell lines were also treated with conditioned medium from poly(I:C)-transfected cells in order to assess their ability to respond to exogenously administered IFN that was contained in the medium (Figure 5A).

Neuro2A cells that carried the WT replicon, when treated with IFN-containing medium, presented a diminished ability to respond in comparison to the control cells (Figure 5A). This was expected since the inhibitory effect of WNV on the cell’s immune response has already been demonstrated in other cell lines. Multiple steps of the antiviral signaling are attenuated by viral factors such as the nonstructural proteins and the sfRNAs that are being produced [40,41,42,43]. However, IFN response levels in sfRNA1-deficient replicon cell line were also significantly low (Figure 5A). Despite the upregulation in a number of innate immunity-related genes that was observed in the differential expression analysis (Ifih1 Cxcl1, Ifit1, Mx2, Irf9) (Figure 3E), it seems that they had minimal effect on IFN competency. Upregulation of such genes should also lead to a reduced replication efficiency of a virus. However, when ΔsfRNA1 replicon cells were infected with WT WNV, they produced comparable viral titer to that of WT replicon cells, meaning that their biological significance on virus replication was also non-significant (Figure 5B).

On the other hand, the WNV-infected ΔsfRNA2 replicon cell line produced a higher viral titer than that of the ΔsfRNA1 and WT replicon cell lines (Figure 5B). In another experiment, ΔsfRNA2 cells that were cleared from the replicon via ribavirin treatment (ΔsfRNA2 CURED) in order to eliminate its inhibitory effect produced an even higher viral titer than that of ΔsfRNA2 cells, almost reaching the levels of plain Neuro2A cells (Figure 5C). These results further support the notion that antiviral innate immunity pathways are downregulated in the ΔsfRNA2 replicon cell line. This state compensates for the replicon’s double sfRNA deficiency that would normally result in its elimination by the activated IFN response. Additionally, the fact that these inherently IFN-incompetent cells presented comparable levels of IFN response to the WT replicon cells proves once again the strong inhibitory effect of both sfRNAs on interferon signaling (Figure 5A).

## 4. Discussion

WNV is a neurotropic flavivirus and one of the most common causes of encephalitis in the world. Up until now, most research regarding sfRNA biosynthesis has been conducted on the commonly used mammalian or mosquito cell lines. Another issue is that the primary focus of these studies is the identification of the sequences that form the tertiary structures for XRN1 stalling [16,17,44,45]. In our study, we investigated the biosynthesis of sfRNAs from a WNV lineage II replicon and their importance for WNV replication in a neuronal cell line. We identified for the first time in neuronal cells, the precise starting points on the viral genome for three sfRNA species using a novel pulldown methodology and next-generation sequencing. The sizes of these sfRNAs were estimated at 512 bp (sfRNA1), 365 bp (sfRNA2), and 271 bp (sfRNA3). Similar results were found by Zhang et al., who sequenced the sfRNAs isolated from WNV-infected Aedes albopictus C6/36 cells [44]. In order to investigate the production of sfRNA4 in neuronal cells, we also constructed a ΔsfRNA3 replicon. However, this triple deficiency in sfRNA production resulted in severe attenuation of the replicon.

In assessing the requirement of sfRNAs in WNV lineage II replication in neuronal cells, we found that sfRNA1 was redundant for the both replication and translation of the WNV replicon in the Neuro2A cell line (Figure 2A,B). Studies in the other cell types are also in support of this notion, as sfRNA1-deficient viral mutants in BHK cells had similar replication levels to those of WT at 48 h post-electroporation. The same mutants demonstrated normal translation efficiency at 4 h post-electroporation [16]. In another study which used an sfRNA1-deficient WNV, the mutant virus presented similar replication kinetics in Vero cells as those of the WT virus, with a comparable CPE effect [45]. The same redundancy effect was recapitulated in mosquito cells in which sfRNA1 was not required for efficient WNV replication [45]. Studies on other flaviviruses (DENV, YFV, ZIKV) and even insect-specific flaviviruses (ISFs) are also in accordance with the notion that sfRNA1 is functionally redundant for viral replication [17,46,47,48,49,50,51]. However, sfRNA1 seems to play a very important role for the manifestation of viral pathogenicity and cytopathicity [16,17,48,51].

The lack of both sfRNA1 and sfRNA2 resulted in significant reduction in both replication and translation efficiency (Figure 2A,B). Employing a selection medium, we managed to generate a Neuro2A cell line that sustained the replication of the ΔsfRNA2 replicon, as verified by negative strand RNA synthesis (Figure 2C). The ΔsfRNA2 replicon cell line presented similar levels of WNV replicon RNA which was in contrast to the deficiency of the ΔsfRNA2 replicon in transient transfection. Differential mRNA expression analysis showed that the ΔsfRNA2 replicon cell line showed downregulation in a significant number of genes implicated in interferon signaling, not only versus ΔsfRNA1 replicon cell line but also versus control cells. An impairment in the innate immunity response of the ΔsfRNA2 replicon cell line could explain the increased efficiency in sustaining the ΔsfRNA2 replicon. Testing this cell line for its infectability by WNV, we observed a trend of increased virus production by the ΔsfRNA2 replicon cell line that was possibly enhanced by the reduced competence of the cells regarding innate immunity, although other pathways may also contribute to it (Figure 5B).

The mechanism of sfRNA action remains unclear. There are multiple sources regarding the inhibitory effect of flaviviral sfRNA on IFN signaling and ISG expression [15,18,19,20,43,51,52,53,54]. However, to our best knowledge, there are no studies proposing distinct roles for sfRNA1 and sfRNA2. In most of the cases, high sensitivity to the antiviral effects of IFNs exhibit the viral mutants that are deficient in both sfRNAs [43,51]. Regardless of this, sfRNA1 has been shown to bind to some mediators of IFN signaling (TRIM25, G3BP1, G3BP2, and CAPRIN1) [19,20,55]. In our differential expression analysis, the ΔsfRNA1 replicon cell line showed upregulation in the expression of some genes related to interferon and interleukin signaling, such as viral RNA sensors (RIG-I, MDA5) and pro-inflammatory cytokines and chemokines (Il7, Cxcl1, and Cxcl10) (Figure 3). However, as sfRNA1 could efficiently replicate after transient transfection and as the ΔsfRNA1 replicon cell line produced the same WNV titer as did the WT replicon cell line (Figure 5B), we can conclude that the differentially expressed genes had minimal effect on virus replication. It seemed that although the lack of sfRNA1 in Neuro2A replicon cells led to some significant differences in the expression of innate immunity genes, their biological significance on virus replication was minimal.

Among the upregulated genes in ΔsfRNA1 replicon cells, Npy and Tmsb4x were significantly upregulated (Figure 3E). Neuropeptide Y (NPY) is a pleiotropic signaling molecule that regulates many physiological processes [56]. A protective role has been proposed for NPY regarding neurodegenerative/neuroimmune disorders and viral infections since it regulates calcium homeostasis, reduction of oxidative stress, protection from excitotoxic cell death, and neuroinflammation attenuation [57,58,59,60,61]. TMSB4X is widely expressed in the neurons and microglia, exerting a neuroprotective effect as an anti-inflammatory effector [62]. Treatment with TMSB4X has been shown to promote recovery in the case of neural injury or neurodegenerative disease, as well as the survival of neurons [63,64,65]. Both of these genes, along with Fgfr3 and Wnt9a, which were also tested, seem to be potential targets of WNV sfRNA1. Wnt-signaling has also been identified as a target of ZIKV sfRNA. Gene ontology enrichment analysis has demonstrated that ZIKV sfRNA inhibits the expression of Wnt7a, leading neural cells to apoptosis and also affecting brain development due to the shared role of Wnt-signaling in both processes [54].

The ΔsfRNA2 replicon cell line presented a different network of differentially regulated genes as a result of the absence of both sfRNAs but also the selection process (Figure 3). As already mentioned, the overall antiviral response of the ΔsfRNA2 replicon cell line was weakened, probably in order to sustain the replication of the attenuated ΔsfRNA2 replicon. In addition to this, genes implicated in Ca^2+^ signaling pathways were also found to be upregulated. Ca^2+^ signaling has proven to be very important for the efficient replication of WNV during the early stages of the infection. It seems that WNV promotes a rapid and sustained Ca^2+^ influx [66]. Gspt2 gene expression levels, although increased in ΔsfRNA1, were further increased in ΔsfRNA2. GSPT2/eRF3b is a GTPase that forms a complex with eRF1, and together, they regulate the termination of translation and possibly mRNA stability. eRF3 has a proteolytically processed isoform (p-eRF3) that binds to the inhibitor of apoptosis proteins (IAPs), such as survivin, and promotes apoptosis [67,68]. It has also been proposed that p-eRF3 localizes in the nucleus where it interacts with the ARF tumor suppressor, possibly mediating pro-apoptotic signals to the nucleus and providing an additional step to the regulation of cell death [69]. It is possible that sfRNA downregulates its expression in order to inhibit apoptosis and ensure the survival of the virus.

As our procedure incorporated a step of selection of the cells that could efficiently sustain WNV replicon, we analyzed the differential expression of genes between replicon cell lines (WT and ΔsfRNA) and the control Neuro2A cells in order to identify transcriptional changes that support viral replication. A selection procedure using hepatitis C virus (HCV) replicons in the past resulted in the generation of cell line Huh7-lunet from the Huh7 line that could efficiently sustain HCV replication [70]. Previous differential expression analyses in WNV-infected neuronal tissues have given some insights into the mechanisms contributing to viral pathogenesis (excessive antiviral immune response, upregulated cell death pathways, nucleic acid editing and repair, protein degradation, glutamate excitotoxicity, decrease in dopamine signaling, defective mitochondrial function) [71,72,73,74,75,76,77,78]. In our case, TCA cycle and oxidative phosphorylation (OXPHOS) were significantly upregulated in all replicon cell lines (Figure 4). An enhanced energy has been shown to be stimulated by type I IFN in order to support the high energy demands for the synthesis of the effectors that orchestrate an effective antiviral response [79,80,81]. A consistent drop in three mitochondrial NADH dehydrogenase subunits that was observed in ΔsfRNA1 replicon cells reverted to WT levels in ΔsfRNA2 (Figure 3E) either due to the attenuated phenotype of the replicon or the transition of the cells toward more supportive energy production levels.

Cell cycle checkpoint control mechanisms/DNA repair pathways were also upregulated in the replicon cell lines. WNV replication also seems to positively regulate post-translational modification processes that include the tagging of small regulatory molecules into target proteins (neddylation, SUMOylation, ubiquitination). Ubiquitin–proteasome regulation has previously been shown to be important for flaviviruses [82,83,84], as well as the reverse process of deubiquitination [85]. Ubiquitin-specific peptidase 18 (USP18), especially, was significantly overexpressed in the WT and ΔsfRNA1 replicon cell lines. USP18 is an isopeptidase, known for its suppression of the IFN signaling pathway [86]. It draws the cell into a non-responsive state, with reduced sensitivity to future stimulation that assists in viral replication [87,88]. This IFN resistance due to increased expression of USP18 has previously been observed in DENV infection [89]. It is also worth noting that receptor transporter protein 4 (RTP4) presented the most significantly high upregulation in all the Neuro2A replicon cell lines. RTP4 has been previously characterized as a negative regulator of the IFN-I response and is associated with higher WNV load in the brains of infected mice [90].

The differential expression analysis of replicon cell lines also identified a set of negatively regulated genes in comparison with the control cell line. Nervous system development, axon guidance, and autophagy were significantly downregulated in all three replicon Neuro2A cell lines. Although no link between developmental processes and WNV has been reported in previous studies, differentiation pathways may be activated during selection as modifiers of the cell identity toward a more permissive cell line; for example, the Sonic hedgehog pathway in Huh7.5 cells stimulates HCV replication [91]. Autophagy during WNV replication, on the other hand, has always been a controversial issue [92]. The autophagic pathway is usually upregulated in order to eliminate the invading virus and to activate the adaptive immune system by presenting its antigens [93]. In other cases, however, autophagy can enhance the propagation of a virus by helping it escape from the cell’s immune response or release its infectious particles [94]. Most studies attribute a non-significant role to autophagy for WNV replication, whether it is activated or not during the infection [95,96]. More recently, however, Kobayashi et al. showed that the accumulation of ubiquitinated proteins from the inhibition of autophagy caused by WNV could be the primary reason for the development of neurological symptoms [97]. Autophagy was also negatively regulated in our replicon neuroblastoma cells.

Gene enrichment analysis also showed that the mTOR signaling pathway was dysregulated in replicon cell lines. Both positive and negative regulators of mTOR were found among the significantly downregulated genes. mTOR protein complexes play a critical role in a number of cellular processes such as cell growth and proliferation, cell survival, transcription, protein synthesis, and autophagy. It is known that WNV activates mTOR in order to maintain the translation of its RNA genome and delay apoptosis [98,99,100]. However, TP53 transcriptional regulation, which has an opposing role in cell growth processes regulated by mTORC1, was also upregulated [101,102,103,104]. A downregulation was also observed in the expression levels of a number of Rho GTPases, G protein-signal transducers, and related regulatory proteins. Proteins of this family are responsible for the regulation of the movement of the cell and cytoskeletal dynamics. WNV itself is known to use clathrin-mediated endocytosis in order to enter into the host cell, explaining why Rho-GTPases as well as other G proteins were previously found to be upregulated in WNV-infected mouse brain [105,106]. In our study, however, Rho GTPase signaling was negatively regulated, possibly demonstrating the deficit in axonal growth which normally requires rearrangement of the cytoskeleton. A deficit in Ca^2+^ signaling was also observed. GTPase signaling has also been shown to be regulated by Ca^2+^ in certain developmental processes, such as axon guidance and neuronal migration [107,108].

## 5. Conclusions

In conclusion, in this study, we investigated the WNV lineage II sfRNAs’ starting positions in a neuronal cell line using a novel affinity pulldown NGS-based methodology. The requirement of the production of these sfRNAs in the replication and the modulation of gene expression patterns is linked to the sustained replication of sfRNA-deficient replicons. sfRNA1 was dispensable in WNV replication, while the concomitant deletion of sfRNA1 and sfRNA2 drastically diminished replication. Investigating the transcriptional changes during WNV replicon establishment in the neuronal cells in the absence of sfRNAs highlighted the importance of various immune and inflammatory processes as well as several neuronal-specific pathways. Both immunity- and differentiation-related pathways may lead us to the development of a better cell line to support sfRNA-deficient WNV, as these viruses are among the most prominent in WNV vaccine development.

## Data Availability

The data used in this study are presented in the Results section and also Supplementary Materials.

## Supplementary Materials

The following supporting information can be downloaded at https://www.mdpi.com/article/10.3390/v16050812/s1: Tables S1 and S2: Primers used in the study; Table S3: ΔsfRNA1 vs. WT upregulated genes; Table S4: ΔsfRNA1 vs. WT downregulated genes; Table S5: ΔsfRNA2 vs. WT upregulated genes; Table S6: ΔsfRNA2 vs. WT downregulated genes; Tables S7–S11: ΔsfRNA vs. WT pathways; Tables S12 and S13: RT-PCR validation; Table S14: WT vs. control upregulated genes; Table S15: WT vs. control downregulated genes; Table S16: ΔsfRNA1 vs. control upregulated genes; Table S17: ΔsfRNA1 vs. control downregulated genes; Table S18: ΔsfRNA2 vs. control upregulated genes; Table S19: ΔsfRNA2 vs. control downregulated genes; Table S20: Replicon vs. control significant pathways (all); Supplementary Figures S1 and S2. All high-resolution figures can be found at https://www.doi.org/10.6084/m9.figshare.25488589 (accessed on 19 May 2024).
