# Supplementary material for: West Nile Virus Subgenomic RNAs Modulate Gene Expression in a Neuronal Cell Line
Source: Viruses. 2024 May 20;16(5):812. doi: 10.3390/v16050812 (PMC11125720; doi:10.3390/v16050812)
Supplement: Supplementary file 1 [file viruses-16-00812-s001.zip › Supplementary Figures s1, s2.pdf]

A

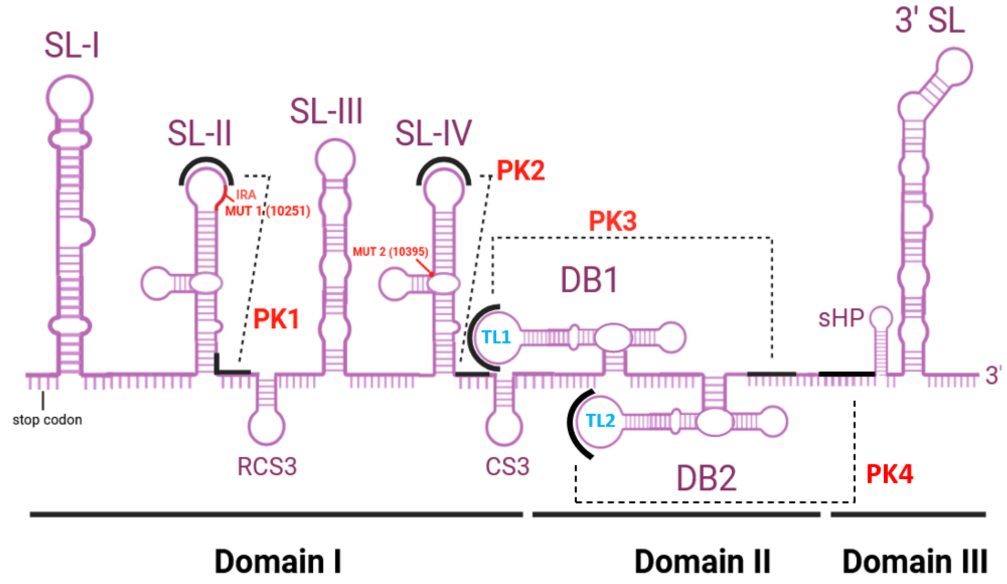

B

WT

10240 10250 10260 10270 10370 10380 10390 10400

gaagttgagtagacggtgctgcctgcggctc |………| cagtgtcagaccacactttaatgtgccactc

ΔsfRNA1

10240 10250 10260 10270 10370 10380 10390 10400

gaagttgagtacaggctgctgcctgcggctc |………| cagtgtcagaccacactttaatgtgccactc

ΔsfRNA2

10240 10250 10260 10270 10370 10380 10390 10400

gaagttgagtacaggctgctgcctgcggctc |………| cagtgtcagaccacactttaatgtggcactc

Supplementary Figure 1. Structural elements within the 3' UTR of WNV and the introduced mutations. (A) Schematic representation of the stem-loops (SL) and pseudoknots (PK) of WNV 3' UTR. The formation of pseudoknots (PK1-4) is depicted by black dashed lines. The position of mutations (MUT1, MUT2) are also indicated in parenthesis. SL-I – SL-IV, stem-loop I – stem loop IV; (R)CS3, short, conserved hairpins; DB1/2, dumbbell 1/ 2; sHP, short hairpin; 3' SL, 3' stem-loop; IRA, inverted repeat A. (B) Nucleotide positions of the introduced mutations on the wild-type (WT) WNV replicon (pCMVWRep2aH-REN), provided by Pierson et al. [21]. ΔsfRNA1 replicon was generated by the substitution of 3 nucleotides in the WT replicon. ΔsfRNA2 replicon was generated by an additional C→G point mutation in the ΔsfRNA1 replicon. Colored regions show the location of introduced mutations.

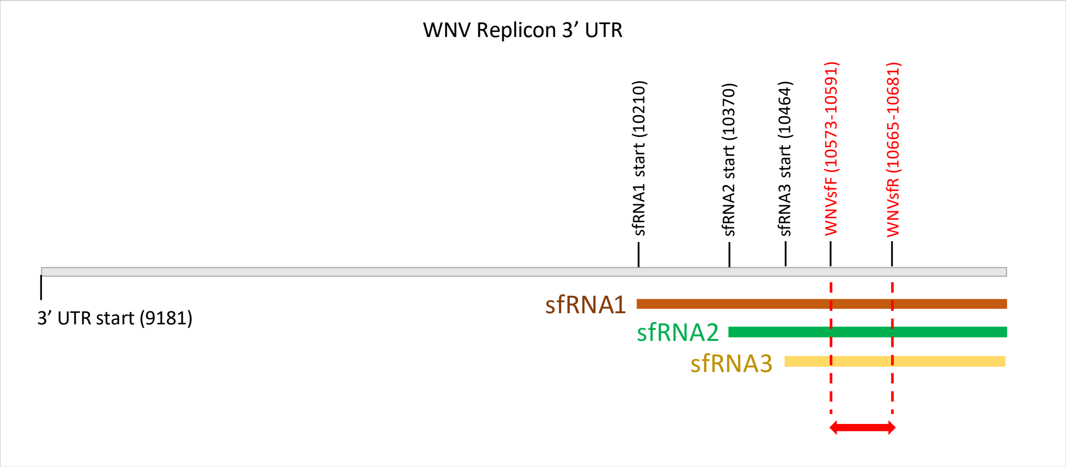

Supplementary Figure 2. Schematic representation of sfRNA-specific primer binding sites. The figure illustrates the 3' UTR of WNV replicon and the starting points of sfRNA species. The nucleotide positions of the primers that target sfRNA (WNVsfF, WNVsfR) are indicated with red dashed lines. The sequences of the sfRNA-specific primers (WNVsfF, WNVsfR) are described in Table S1. As shown, the region targeted by these primers is common for all three sfRNA species. The RT-qPCR analysis and the subsequent procedure for the estimation of the relative abundance of sfRNA species in each replicon cell line, are described at section 2.8.
