# Supplementary material for: West Nile Virus Subgenomic RNAs Modulate Gene Expression in a Neuronal Cell Line
Source: Viruses. 2024 May 20;16(5):812. doi: 10.3390/v16050812 (PMC11125720; doi:10.3390/v16050812)
Supplement: Supplementary file 1 [file viruses-16-00812-s001.zip › Tables S12-S13 RT-PCR validation.pdf]

Table S12. Comparison of the fold change estimates from NGS and real-time qPCR for a selected set of genes that were determined from the NGS analysis as differentially expressed among ΔsfRNA1 and WT replicon stable cell lines.

	**ΔsfRNA1vsWT (fold difference)**	
	NGS	qPCR
**Npy**	91.6	125.3
**Tmsb4x**	77.9	49.5
**Cxcl1**	74.9	5.4
**Gspt2**	62.2	18.2
**Wnt9a**	49.4	3.2
**Ifih1**	6.5	2.9
**Fgfr3**	4.5	1.9
**Cacna1c**	3.2	1.3
**Tubb6**	3.1	2.0
**Ank3**	1.6	1.8
**Pmp22**	1.6	1
**Arrb1**	0.36	0.5
**Sctr**	0.35	0.4
**ND1**	0.32	0.4
**ND4**	0.31	0.5
**Shc1**	0.24	0.2
**ND2**	0.23	0.4

Table S13. Comparison of the fold change estimates from NGS and real-time qPCR for a selected set of genes that were determined from the NGS analysis as differentially expressed among ΔsfRNA2 and WT replicon stable cell lines.

	**ΔsfRNA2vsWT (fold difference)**	
	NGS	qPCR
**Gspt2**	115.8	34.1
**Sox6**	23.1	7.8
**Adam22**	20.7	18.1
**Ank3**	4.3	3.1
**Neurl1b**	6	12.5
**Cacna1c**	3.5	1.6
**Tubb6**	1.7	1.5
**Pmp22**	1.6	1.5
**Trim25**	0.46	0.6
**Shc1**	0.4	0.5
**Arrb1**	0.38	0.7
**Stat1**	0.38	0.2
**Sctr**	0.34	0.8
**Irf9**	0.31	0.4
**Ifit1**	0.08	0.1
**Mx2**	0.07	0.15
