# Supplementary material for: West Nile Virus Subgenomic RNAs Modulate Gene Expression in a Neuronal Cell Line
Source: Viruses. 2024 May 20;16(5):812. doi: 10.3390/v16050812 (PMC11125720; doi:10.3390/v16050812)
Supplement: Supplementary file 1 [file viruses-16-00812-s001.zip › Tables S1-S2 Primers used in the study.pdf]

Table S1. Primers used in the study

Primer name	Sequence (5’-3’)	Construct
WNVIRAmutF	TGAGTA**C**A**G**G**C**TGCTGCCTGCGGCTCAACC	ΔsfRNA1
WNVIRAmutR	GCAGCA**G**C**C**T**G**TACTCAACTTCCGGTGGCA	ΔsfRNA1
WNVIRAextF	CTGAATAGGTGACCGGAGGT	ΔsfRNA1
WNVIRAextR	ACACTGGGCTTTGAGGCTAA	ΔsfRNA1
WNVmut10395F	TAATGTG**G**CACTCTGCGGAG	ΔsfRNA2
WNVmut10395R	CTCCGCAGAGTG**C**CACATTA	ΔsfRNA2
WNVext10395F	GGTTTCCCCAATGACACAAA	ΔsfRNA2
WNVext10395R	GAAGGAGCTGACTGGGTTGAA	ΔsfRNA2
NS1RT4F	GACTGGTGCTGCAGAAGTTG	-
NS1RT4R	GAGGGTCTTTTCGTCGTGC	-
WNVsfF	GCCAAGGGAAGGACTAGAG	-
WNVsfR	CTGGTTGTGCAGAGCAG	
BIO-WNRTRe	[BIO-TEG] CGGTWYTGAGGGCTTACRTGG	-
BIO-WNRTReb	[BIO-TEG] GGGGTCTCCTCTAACCTCTAGTCCTT	-
(mouse)YWHAZF	GAAAAGTTCTTGATCCCCAATGC	-
(mouse)YWHAZR	TGTGACTGGTCCACAATTCCTT	-

Table S2. Primers used in the Differential Expression Analysis

Primer name	Sequence (5'-3')
mADAM22F	GGAGTCGCCTCACTGAAAGG
mADAM22R	CCGCCCAAGCGATAGATGA
mIFIH1F	AGATCAACACCTGTGGTAACACC
mIFIH1R	CTCTAGGGCCTCCACGAACA
mANK3F	ACCAAATACGTGTGGAAAATCCC
mANK3R	GTTCTGCCAACCATCAACTGG
mGSPT2F	CTCTCAAGCCGATTTAGCTGTG
mGSPT2R	ATCCAGTCTCAAATTCCCCTTTC
mNEURL1bF	TATGGCATCACGGACGAAGTA
mNEURL1bR	AGCTCGTTGTTGTCGAAGTTG
mNPYF	ATGCTAGGTAACAAGCGAATGG
mNPYR	TGTCGCAGAGCGGAGTAGTAT
mFGFR3F	GCCTGCGTGCTAGTGTTCT
mFGFR3R	TACCATCCTTAGCCCAGACCG
mCXCL1F	CTGGGATTCACCTCAAGAACATC
mCXCL1R	CAGGGTCAAGGCAAGCCTC
mCACNA1cF	CCTGCTGGTGGTTAGCGTG
mCACNA1cR	TCTGCCTCCGTCTGTTTAGAA
mPMP22F	CATCGCGGTGCTAGTGTTG
mPMP22R	AAGGCGGATGTGGTACAGTTC
mSOX6F	GGTCATGTTTCCCACCCACAA
mSOX6R	TTCAGAGGGGTCCAAATTCCT
mSHC1F	AAGTACAACCCACTTCGGAATG
mSHC1R	GAAAGAAGGAACACAGGGTAGTC
mSCTRF	GTGGGCTGTCACCAGACAC
mSCTRR	ATCAGCAGGAGGGTGGACTT
mARRB1F	AAGGGACACGAGTGTTCAAGA
mARRB1R	CCCGCTTTCCCAGGTAGAC
mTUBB6F	GAGAGGATCAGCGTCTACTACA
mTUBB6R	TGTCCGAAGATGAAGTTGTCAG
mTRIM25F	ATGGCTCAGGTAACAAGGGAG
mTRIM25R	GGGAGCAACAGGGGTTTTCTT
mND2F	CCTCCTGGCCATCGTACTCA
mND2R	GAATGGGGCGAGGCCTAGTT
mND4F	CGCCTACTCCTCAGTTAGCCA
mND4R	TGATGTGAGGCCATGTGCGA
mND1F	GCTTTACGAGCCGTAGCCCA
mND1R	GGGTCAGGCTGGCAGAAGTAA
mIFIT1F	CTGAGATGTCACTTCACATGGAA
mIFIT1R	GTGCATCCCCAATGGGTTCT
mMX2F	GAGGCTCTTCAGAATGAGCAAA
mMX2R	CTCTGCGGTCAGTCTCTCT
mIRF9F	GCCGAGTGGTGGGTAAGAC
mIRF9R	GCAAAGGCGCTGAACAAAGAG
mSTAT1F	TCACAGTGGTTCGAGCTTCAG
mSTAT1R	GCAAACGAGACATCATAGGCA
mTMSB4XF	ATGTCTGACAAACCCGATATGGC
mTMSB4XR	CCAGCTTGCTTCTCTTGTTCA
mWNT9aF	GGCCCAAGCACACTACAAG
mWNT9aR	AGAAGAGATGGCGTAGAGGAAA
