# Supplementary material for: West Nile Virus Subgenomic RNAs Modulate Gene Expression in a Neuronal Cell Line
Source: Viruses. 2024 May 20;16(5):812. doi: 10.3390/v16050812 (PMC11125720; doi:10.3390/v16050812)
Supplement: Supplementary file 1 [file viruses-16-00812-s001.zip › Tables S7-S11 âsfRNA vs WT pathways.pdf]

*Table S7. The following table shows the top 25 Reactome pathways enriched in genes upregulated in ΔsfRNA1 replicon cell line, sorted by Entities ratio (pValue<0.05).*

| Pathway name | Entities | | | Reactions |
|---|---|---|---|---|
| | Found | pValue | FDR | Found |
| Circadian Clock | 12/105 | 2.23E-05 | 2.26E-02 | 26/59 |
| Interleukin-10 signaling | 6/86 | 2.44E-02 | 5.26E-01 | 2/15 |
| Transcriptional activation of mitochondrial biogenesis | 6/88 | 2.68E-02 | 5.26E-01 | 17/32 |
| Nuclear Events (kinase and transcription factor activation) | 6/80 | 1.79E-02 | 5.26E-01 | 28/48 |
| NGF-stimulated transcription | 5/56 | 1.53E-02 | 5.26E-01 | 27/37 |
| PERK regulates gene expression | 5/42 | 4.82E-03 | 5.26E-01 | 6/11 |
| Regulation of Homotypic Cell-Cell Adhesion | 4/39 | 1.86E-02 | 5.26E-01 | 16/33 |
| Assembly and cell surface presentation of NMDA receptors | 4/49 | 3.81E-02 | 5.26E-01 | 6/23 |
| RHO GTPases activate IQGAPs | 5/36 | 2.52E-03 | 5.26E-01 | 5/5 |
| ATF4 activates genes in response to endoplasmic reticulum stress | 4/34 | 1.18E-02 | 5.26E-01 | 3/7 |
| Regulation of CDH11 Expression and Function | 4/35 | 1.30E-02 | 5.26E-01 | 15/28 |
| Transport of connexons to the plasma membrane | 3/23 | 2.18E-02 | 5.26E-01 | 1/3 |
| Post-chaperonin tubulin folding pathway | 3/25 | 2.70E-02 | 5.26E-01 | 6/9 |
| FOXO-mediated transcription of cell cycle genes | 3/27 | 3.27E-02 | 5.26E-01 | 5/22 |
| Formation of tubulin folding intermediates by CCT/TriC | 3/30 | 4.25E-02 | 5.26E-01 | 2/2 |
| TRAF6 mediated NF-kB activation | 3/30 | 4.25E-02 | 5.26E-01 | 3/4 |
| Processing of Capped Intronless Pre-mRNA | 3/30 | 4.25E-02 | 5.26E-01 | 5/10 |
| Signaling by FLT3 fusion proteins | 3/32 | 4.97E-02 | 5.26E-01 | 8/18 |
| Regulation of CDH11 gene transcription | 3/12 | 3.80E-03 | 5.26E-01 | 7/14 |
| FGFR3 mutant receptor activation | 3/17 | 9.83E-03 | 5.26E-01 | 10/10 |
| TP53 Regulates Transcription of Genes Involved in G1 Cell Cycle Arrest | 3/20 | 1.51E-02 | 5.26E-01 | 6/17 |
| STAT5 activation downstream of FLT3 ITD mutants | 3/21 | 1.72E-02 | 5.26E-01 | 13/14 |
| RUNX3 regulates CDKN1A transcription | 2/8 | 1.82E-02 | 5.26E-01 | 3/6 |
| Microtubule-dependent trafficking of connexons from Golgi to the plasma membrane | 3/22 | 1.94E-02 | 5.26E-01 | 1/2 |
| Transcriptional activation of cell cycle inhibitor p21 | 3/6 | 5.31E-04 | 1.79E-01 | 4/5 |

*Table S8. The following table shows the top 25 Reactome pathways enriched in genes downregulated in ΔsfRNA1 replicon cell line, sorted by Entities ratio (pValue<0.05).*

| Pathway name | Entities | | | Reactions |
|---|---|---|---|---|
| | Found | pValue | FDR | Found |
| Signaling by NOTCH | 15/258 | 1.66E-02 | 4.90E-01 | 25/154 |
| The citric acid (TCA) cycle and respiratory electron transport | 16/238 | 3.73E-03 | 4.90E-01 | 16/67 |
| Signaling by NTRKs | 10/166 | 3.69E-02 | 4.90E-01 | 33/164 |
| Respiratory electron transport, ATP synthesis by chemiosmotic coupling, and heat production by uncoupling proteins. | 14/153 | 3.93E-04 | 2.16E-01 | 14/31 |
| Transcriptional Regulation by MECP2 | 7/100 | 3.86E-02 | 4.90E-01 | 12/77 |
| RNA Polymerase I Transcription | 8/86 | 6.06E-03 | 4.90E-01 | 11/14 |
| NoRC negatively regulates rRNA expression | 7/79 | 1.27E-02 | 4.90E-01 | 6/7 |
| RHOG GTPase cycle | 6/78 | 3.66E-02 | 4.90E-01 | 3/6 |
| Retinoid metabolism and transport | 6/79 | 3.85E-02 | 4.90E-01 | 9/28 |
| Transcriptional regulation by small RNAs | 6/80 | 4.05E-02 | 4.90E-01 | 2/5 |
| Complex I biogenesis | 8/59 | 6.07E-04 | 2.16E-01 | 8/13 |
| SUMOylation of chromatin organization proteins | 7/62 | 3.61E-03 | 4.90E-01 | 8/15 |
| Meiotic synapsis | 6/61 | 1.29E-02 | 4.90E-01 | 5/6 |
| HDACs deacetylate histones | 5/63 | 4.84E-02 | 4.90E-01 | 5/5 |
| ERCC6 (CSB) and EHMT2 (G9a) positively regulate rRNA expression | 7/47 | 7.70E-04 | 2.16E-01 | 4/4 |
| Elastic fibre formation | 5/45 | 1.40E-02 | 4.90E-01 | 8/17 |
| RNA Polymerase I Transcription Initiation | 5/50 | 2.10E-02 | 4.90E-01 | 4/6 |
| Assembly of the ORC complex at the origin of replication | 4/39 | 3.45E-02 | 4.90E-01 | 5/11 |
| Recognition and association of DNA glycosylase with site containing an affected pyrimidine | 4/41 | 4.02E-02 | 4.90E-01 | 1/21 |
| Signaling by ALK | 4/43 | 4.65E-02 | 4.90E-01 | 3/40 |
| SIRT1 negatively regulates rRNA expression | 4/44 | 4.97E-02 | 4.90E-01 | 4/5 |
| Cleavage of the damaged purine | 4/44 | 4.97E-02 | 4.90E-01 | 2/9 |
| SMAD2/SMAD3:SMAD4 heterotrimer regulates transcription | 4/44 | 4.97E-02 | 4.90E-01 | 4/24 |
| MECP2 regulates neuronal receptors and channels | 4/32 | 1.85E-02 | 4.90E-01 | 6/26 |
| Negative regulators of DDX58/IFIH1 signaling | 4/37 | 2.93E-02 | 4.90E-01 | 3/13 |

*Table S9. The following table shows the top 25 Reactome pathways enriched in genes upregulated in ΔsfRNA2 replicon cell line, sorted by Entities ratio (pValue<0.05).*

| Pathway name | Entities | | | Reactions |
|---|---|---|---|---|
| | Found | pValue | FDR | Found |
| Signaling by Receptor Tyrosine Kinases | 15/623 | 1.75E-02 | 3.87E-01 | 175/746 |
| Platelet activation, signaling and aggregation | 8/293 | 3.99E-02 | 3.87E-01 | 7/117 |
| Muscle contraction | 7/232 | 3.39E-02 | 3.87E-01 | 14/53 |
| Cardiac conduction | 6/147 | 1.34E-02 | 3.87E-01 | 10/33 |
| Response to elevated platelet cytosolic $Ca^{2+}$ | 6/148 | 1.38E-02 | 3.87E-01 | 2/14 |
| Signaling by VEGF | 6/140 | 1.08E-02 | 3.87E-01 | 52/86 |
| Platelet degranulation | 6/141 | 1.11E-02 | 3.87E-01 | 2/11 |
| Negative regulation of the PI3K/AKT network | 5/137 | 3.50E-02 | 3.87E-01 | 2/10 |
| Regulation of cholesterol biosynthesis by SREBP (SREBF) | 4/87 | 2.81E-02 | 3.87E-01 | 4/52 |
| Constitutive Signaling by Aberrant PI3K in Cancer | 4/96 | 3.81E-02 | 3.87E-01 | 2/2 |
| Signaling by EGFR | 4/61 | 8.80E-03 | 3.87E-01 | 16/49 |
| Signaling by NOTCH3 | 4/63 | 9.81E-03 | 3.87E-01 | 4/37 |
| PI3K Cascade | 3/58 | 4.12E-02 | 3.87E-01 | 2/6 |
| Signaling by FGFR1 | 3/62 | 4.85E-02 | 3.87E-01 | 18/48 |
| Glucagon-like Peptide-1 (GLP1) regulates insulin secretion | 3/50 | 2.85E-02 | 3.87E-01 | 7/12 |
| Signaling by FGFR4 | 3/51 | 2.99E-02 | 3.87E-01 | 18/41 |
| Signaling by FGFR3 | 3/53 | 3.30E-02 | 3.87E-01 | 18/43 |
| Response of EIF2AK1 (HRI) to heme deficiency | 5/29 | 4.55E-05 | 2.84E-02 | 8/20 |
| Signaling by ERBB2 in Cancer | 3/36 | 1.21E-02 | 3.87E-01 | 9/62 |
| NOTCH2 Activation and Transmission of Signal to the Nucleus | 2/23 | 3.69E-02 | 3.87E-01 | 1/11 |
| TNFR1-induced proapoptotic signaling | 2/26 | 4.60E-02 | 3.87E-01 | 1/3 |
| Constitutive Signaling by Ligand-Responsive EGFR Cancer Variants | 2/26 | 4.60E-02 | 3.87E-01 | 3/23 |
| Nuclear events stimulated by ALK signaling in cancer | 2/27 | 4.92E-02 | 3.87E-01 | 1/9 |
| Signaling by MST1 | 2/8 | 5.07E-03 | 3.87E-01 | 4/4 |
| MET activates PI3K/AKT signaling | 2/10 | 7.79E-03 | 3.87E-01 | 5/5 |

*Table S10. The following table shows the top 25 Reactome pathways enriched in genes downregulated in ΔsfRNA2 replicon cell line, sorted by Entities ratio (pValue<0.05).*

| Pathway name | Entities | | | Reactions |
|---|---|---|---|---|
| | Found | pValue | FDR | Found |
| Immune System | 50/2627 | 1.92E-04 | 1.67E-02 | 272/1664 |
| Infectious disease | 27/1445 | 9.44E-03 | 1.35E-01 | 75/897 |
| Cytokine Signaling in Immune system | 36/1039 | 3.37E-09 | 1.33E-06 | 171/745 |
| Signaling by Interleukins | 15/658 | 1.03E-02 | 1.35E-01 | 89/505 |
| Signaling by Receptor Tyrosine Kinases | 16/623 | 2.66E-03 | 9.06E-02 | 119/746 |
| Diseases of signal transduction by growth factor receptors and second messengers | 13/498 | 5.78E-03 | 1.35E-01 | 75/478 |
| Interferon Signaling | 21/322 | 2.59E-10 | 2.05E-07 | 44/79 |
| ESR-mediated signaling | 8/256 | 1.07E-02 | 1.35E-01 | 22/114 |
| Signaling by NOTCH | 8/258 | 1.11E-02 | 1.35E-01 | 26/154 |
| Cellular Senescence | 7/199 | 9.12E-03 | 1.35E-01 | 13/90 |
| G alpha (s) signalling events | 9/190 | 4.28E-04 | 3.38E-02 | 14/28 |
| Signaling by NTRKs | 9/166 | 1.61E-04 | 1.57E-02 | 48/164 |
| Opioid Signalling | 5/113 | 1.08E-02 | 1.35E-01 | 18/59 |
| Antiviral mechanism by IFN-stimulated genes | 7/94 | 1.31E-04 | 1.48E-02 | 12/31 |
| Interleukin-10 signaling | 5/86 | 3.52E-03 | 1.09E-01 | 1/15 |
| ISG15 antiviral mechanism | 7/83 | 6.10E-05 | 9.63E-03 | 11/16 |
| Transcriptional regulation of granulopoiesis | 5/70 | 1.46E-03 | 5.98E-02 | 5/27 |
| RAF activation | 3/41 | 1.25E-02 | 1.35E-01 | 3/12 |
| Negative regulators of DDX58/IFIH1 signaling | 4/37 | 9.85E-04 | 5.12E-02 | 9/13 |
| Growth hormone receptor signaling | 3/29 | 4.90E-03 | 1.35E-01 | 5/28 |
| TRAF6 mediated NF-kB activation | 3/30 | 5.38E-03 | 1.35E-01 | 2/4 |
| Interleukin-6 family signaling | 3/30 | 5.38E-03 | 1.35E-01 | 8/34 |
| CLEC7A/inflammasome pathway | 3/8 | 1.23E-04 | 1.48E-02 | 2/4 |
| Apoptosis induced DNA fragmentation | 3/13 | 5.06E-04 | 3.59E-02 | 2/12 |
| NF-kB activation through FADD/RIP-1 pathway mediated by caspase-8 and -10 | 3/14 | 6.26E-04 | 3.76E-02 | 5/5 |

*Table S11. The following table shows the top 25 Reactome pathways enriched in genes downregulated in both ΔsfRNA1 and ΔsfRNA2 replicon cell lines, sorted by Entities ratio (pValue<0.05).*

| Pathway name | Entities | | | Reactions |
|---|---|---|---|---|
| | Found | pValue | FDR | Found |
| Signaling by GPCR | 5/876 | 4.84E-02 | 5.80E-02 | 7/392 |
| Diseases of signal transduction by growth factor receptors and second messengers | 4/498 | 2.67E-02 | 5.57E-02 | 51/478 |
| Signaling by Nuclear Receptors | 5/386 | 1.80E-03 | 3.60E-02 | 16/196 |
| MAPK family signaling cascades | 4/380 | 1.09E-02 | 4.38E-02 | 9/122 |
| RAF/MAP kinase cascade | 4/322 | 6.20E-03 | 3.84E-02 | 9/75 |
| Deubiquitination | 3/289 | 2.85E-02 | 5.69E-02 | 3/77 |
| ESR-mediated signaling | 5/256 | 2.86E-04 | 1.82E-02 | 16/114 |
| Signaling by NOTCH | 3/258 | 2.12E-02 | 5.57E-02 | 5/154 |
| Ub-specific processing proteases | 3/206 | 1.17E-02 | 4.39E-02 | 3/40 |
| G alpha (s) signalling events | 5/190 | 7.18E-05 | 8.33E-03 | 6/28 |
| Signaling by NTRKs | 3/166 | 6.53E-03 | 3.84E-02 | 13/164 |
| Unfolded Protein Response (UPR) | 2/156 | 4.99E-02 | 5.80E-02 | 1/99 |
| Homology Directed Repair | 2/130 | 3.60E-02 | 5.80E-02 | 2/52 |
| Signaling by FGFR | 2/107 | 2.52E-02 | 5.57E-02 | 16/142 |
| IGF1R signaling cascade | 3/72 | 6.17E-04 | 1.93E-02 | 5/17 |
| DNA Double Strand Break Response | 2/75 | 1.30E-02 | 4.39E-02 | 29/48 |
| RHO GTPases activate PKNs | 2/79 | 1.43E-02 | 4.39E-02 | 8/20 |
| Signaling by ERBB2 | 3/68 | 5.23E-04 | 1.93E-02 | 6/46 |
| SHC1 events in ERBB2 signaling | 3/36 | 8.16E-05 | 8.33E-03 | 6/6 |
| Interleukin-2 signaling | 1/14 | 3.17E-02 | 5.80E-02 | 2/19 |
| Interleukin-15 signaling | 1/16 | 3.61E-02 | 5.80E-02 | 6/17 |
| IL-6-type cytokine receptor ligand interactions | 1/17 | 3.83E-02 | 5.80E-02 | 2/14 |
| SHC1 events in EGFR signaling | 1/19 | 4.27E-02 | 5.80E-02 | 4/4 |
| IRF3-mediated induction of type I IFN | 1/19 | 4.27E-02 | 5.80E-02 | 3/5 |
| Regulation of innate immune responses to cytosolic DNA | 1/21 | 4.71E-02 | 5.80E-02 | 4/9 |
